# Enhance photoelectrochemical hydrogen-generation activity and stability of TiO_2_ nanorod arrays sensitized by PbS and CdS quantum dots under UV-visible light

**DOI:** 10.1186/s11671-015-1129-3

**Published:** 2015-10-26

**Authors:** Lei Li, Haitao Dai, Liefeng Feng, Dan Luo, Shuguo Wang, Xiaowei Sun

**Affiliations:** Tianjin Key Laboratory of Low Dimensional Materials Physics and Preparing Technology, School of Science, Tianjin University, Tianjin, 300072 China; Department of Electrical and Electronic Engineering, South University of Science and Technology of China, Shenzhen, 518055 China; Nanyang Technological University, School of Electrical and Electronic Engineering, LUMINOUS! Centre of Excellence for Semiconductor Lighting and Displays, Singapore, 639798 Singapore

**Keywords:** Photoelectrochemical, Solar water splitting, Hydrogen generation, 8200008245+z, 8250Fv

## Abstract

We develop a composite photoanode by sensitizing TiO_2_ nanorod arrays with PbS quantum dots (QDs) and CdS QDs. Benefitted from additional introduced PbS QDs and CdS QDs onto TiO_2_, the absorption of the composite photoanodes are broaden from UV to visible region. The experimental results showed that the PbS sandwiched between TiO_2_ and CdS cannot only broad the absorption properties but also improve the stability. The stability can be explained by the hole facile transmission from PbS to CdS because of the valence band offsets between PbS and CdS which cause a small energy barrier and reduce the hole accumulation. The photocurrent density reached 1.35 mA cm^−2^ at 0.9716 V vs. RHE (0 V vs. Ag/AgCl, under 60 mW cm^−2^ illumination) for TiO_2_/PbS/CdS. The highest photocurrent of TiO_2_/PbS/CdS can be explained by the smallest of total resistance (138 Ω cm^−2^) compared to TiO_2_/CdS and pristine TiO_2_.

## Background

Energy crisis is one of the great challenges of the twenty-first century facing humankind due to the excessive dependence on fossil fuels. Solar energy, as a renewable and almost inexhaustible energy, is expected as one promising candidate to resolve the upcoming crisis. Solar energy can be utilized via a variety of fashions, such as solar cells [1], photoelectrochemical (PEC) device for hydrogen production [2], thermal energy storage [3], and so on [4]. Hydrogen energy, as a clean energy, has been a promising candidate for next-generation energy. Especially, after Fujishima and Honda found the direct hydrogen generation by solar water splitting with TiO_2_ photoanode in 1972, PEC cells based on TiO_2_ for solar hydrogen production have been studied extensively [5–9]. This method paves a way to generate clean hydrogen energy by means of almost inexhaustible solar energy to split water (most abundant materials in Earth) mediated with wide bandgap semiconductor TiO_2_ (suitable bandgap, chemical stability, cost effectiveness, and environmental friendliness [10, 11]). However, the performance of devices based on wide bandgap semiconductor (ZnO, TiO_2_, etc.) is limited by the narrow absorption range. For TiO_2_ (3.0 eV for rutile TiO_2_, 3.2 eV for anatase TiO_2_), only UV light, which carries about 4 % power of sunlight, can be effectively utilized which also limits the performance of PEC for hydrogen generation. To enhance or broaden the absorption region of TiO_2_, massive methods have been explored, for example, introducing proper dopants [10–13] and increasing specific surface area [14]. Among them, sensitizing the narrow bandgap semiconductor such as CdS [15–19], CdSe [13, 15, 16], Bi_2_S_3_ [20], PbS [17, 21, 22], and CdTe [23] with TiO_2_ to broaden the visible absorption is emerging as an effective method. Narrow bandgap mental sulfide, such as CdS and PbS, has been investigated comprehensively for application in solar-to-hydrogen due to the considerable absorption in visible and near-infrared spectrum [24–30]. However, narrow bandgap of PbS lead oxidation of S^2−^ attributed to hole accumulation, which causes photo-corrosion and decreases the stability of devices [26, 31].

In the present work, we prepared the heterojunction of PbS QDs and CdS QDs by means of the successive ionic layer absorption and reaction (SILAR) process on TiO_2_ nanorod arrays. Experimental results show that fabricated photoanode based on TiO_2_ nanorod arrays sensitized with the PbS/CdS heterojunction could improve UV-visible absorption and boost the photocurrent density. Meanwhile, due to valence band offsets between PbS and CdS, the hole accumulation is reduced, which improved the stability of TiO_2_/PbS/CdS photoanode.

## Methods

### Preparation of Photoanodes

TiO_2_ nanorod arrays were prepared on FTO glass initially based on conventional solvent-based method [20]. The cleaned FTO substrate was placed upside down in a sealed Teflon reactor filled with hydrochloric acid (15 mL), deionized water (15 mL), and titanium *n*-butoxide (0.5 mL) at 150 °C for 8 h. After the reaction, the FTO substrate was taken out, rinsed with deionized water, and dried in ambient air. To sensitize PbS and CdS QDs, the FTO substrate covered with TiO_2_ nanorod arrays were sequentially dipped into various precursor solutions (Cd(Ac)_2_, Pb(Ac)_2_, and Na_2_S). First, the TiO_2_ nanorods with FTO substrate were immersed into 0.02 M methanolic solution of lead acetate (Pb(Ac)_2_) and 0.02 M solution of Na_2_S · 9H_2_O in methanol-water (1:1, *v*/*v*). After rinsing with methanol, PbS QDs are sensitized on TiO_2_ nanorods. At last, the substrate is dipped into the 0.05 M precursor solution of Cd^2+^ and S^2−^ to prepare CdS QDs.

### Characterization

In our experiments, three samples are prepared, i.e., pristine TiO2, TiO_2_/CdS, and TiO_2_/PbS/CdS, for performance comparison. The morphologies are observed with a field-emission scanning electron microscopy (SEM, Hitachi, S-4800) and transmission electron microscopy equipped with an energy-dispersive X-ray spectroscopy (EDS) (TEM, JEM-2100F). The UV-visible absorption spectra are measured with a spectrometer (UV-3600, Shimadzu) under diffuse reflection method. The crystalline phase was recorded by X-ray diffraction (XRD) patterns, with a two theta value range from 10° to 90°, at 5°/min (Rigaku, D/MAX-2500). X-ray photoelectron spectroscopy (XPS) was measured on Thermo Scientific Theta Probe XPS.

### Evaluation PEC Performance

The PEC properties are studied with an electrochemical workstation (CHI660D) with a three-electrode system in 0.35 M Na_2_SO_3_ and 0.25 M Na_2_S (PH = 13) electrolyte solution at room temperature. The substrate, Pt mesh, and Ag/AgCl electrode are used as working electrode, counter electrode, and reference electrode, respectively. A 150 W Xenon light source with AM1.5 filter is used to irradiate to the substrate, and the power of the solar simulator is measured to be 60 mW · cm^−2^. The linear sweep voltammetry (LSV) is recorded at a scan rate 10 mV/s, with chopped AM1.5G simulated sunlight irradiation. *I–t* curves were measured at 0.9716 V vs. RHE under AM1.5 irradiation. Electrochemical impedance spectroscopy (EIS) was measured from 1 Hz to 100 kHz, with AC amplitude of 5 mV. IPCE measurements were taken with a tungsten light and monochromator (a standard silicon cell as a reference).

## Results and Discussion

**Fig. 1:**
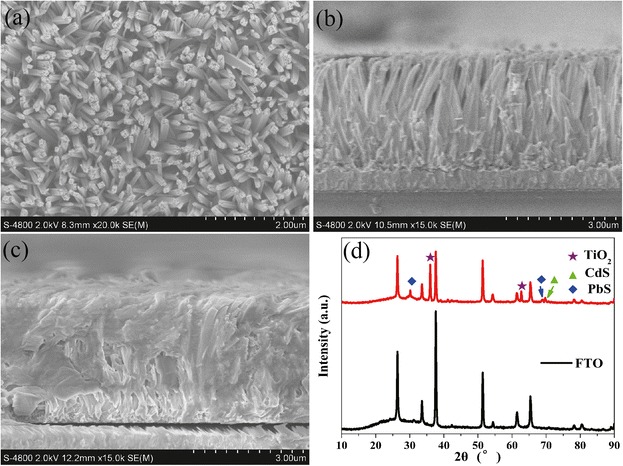
The top (**a**) and cross-section (**b**) FESEM images of pristine TiO_2_. The cross-section FESEM of TiO_2_ (**c**) after depositing PbS and CdS QDS. XRD patterns (**d**) of FTO glass and TiO_2_/PbS/CdS

First, the morphologies, structures, and crystalline phases of the composite system are investigated via FESEM and XRD shown in Fig. 1a–d. According to Fig. 1a, the fabricated TiO_2_ nanorod arrays covered the FTO glass uniformly. The diameter of the TiO_2_ nanorod is about 110 nm. Figure 1b shows the cross-section FESEM image of TiO_2_ nanorod arrays. The length of the TiO_2_ nanorod is about 3 μm. After depositing PbS QDs and CdS QDs, we can see that the QDs cover the nanorod arrays with large area from the cross-section FESEM image shown in Fig. 1c. The XRD patterns of TiO_2_/PbS/CdS are recorded in Fig. 1d. The diffraction peaks corresponding to SnO_2_ are attributed to FTO glass. The diffraction peaks are located at the *2θ* = 36.1° and 62.7° corresponding to rutile TiO_2_ phase. *2θ* = 30.1° and 69.1° corresponding to PbS phase. The diffraction peaks are located at the *2θ* = 70.4° corresponding to CdS phase [32]. The diffraction peaks of CdS phase is not easy to find, because some peaks are located very close to SnO_2_, such as *2θ* = 26.23° and 52.06° [2].

**Fig. 2:**
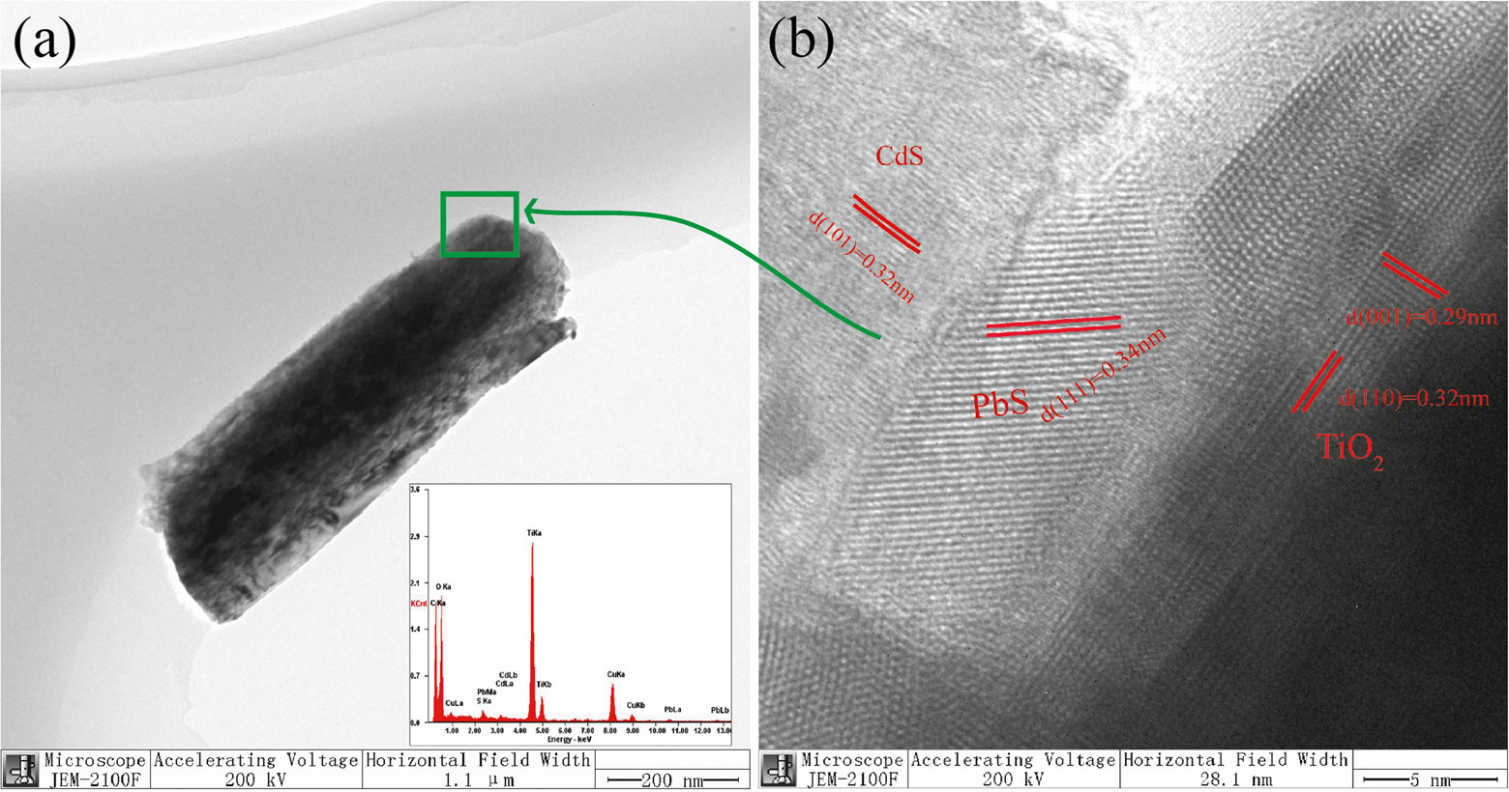
TEM image and EDS (**a**) of TiO_2_/PbS/CdS. HRTEM image (**b**) of marked area in (**a**)

To further prove crystalline phase, the HRTEM and EDS were investigated and shown in Fig. 2. According to inset of Fig. 2a, the EDS of the nanostructure of the Ti, O, Cd, Pb, and S elements are mainly from TiO_2_, CdS, and PbS. C element is from the carbon film of Cu mesh. Figure 2b shows the HRTEM of the marked area in Fig. 2a. The *d*-spacing of (001) and (110) in Fig. 2b is consistent with rutile TiO_2_, which are 0.29 and 0.32 nm, respectively. The *d*(111) = 0.34 nm is consistent with rock salt PbS. The *d*(101) = 0.32 nm of CdS is shown in Fig. 2b.

**Fig. 3:**
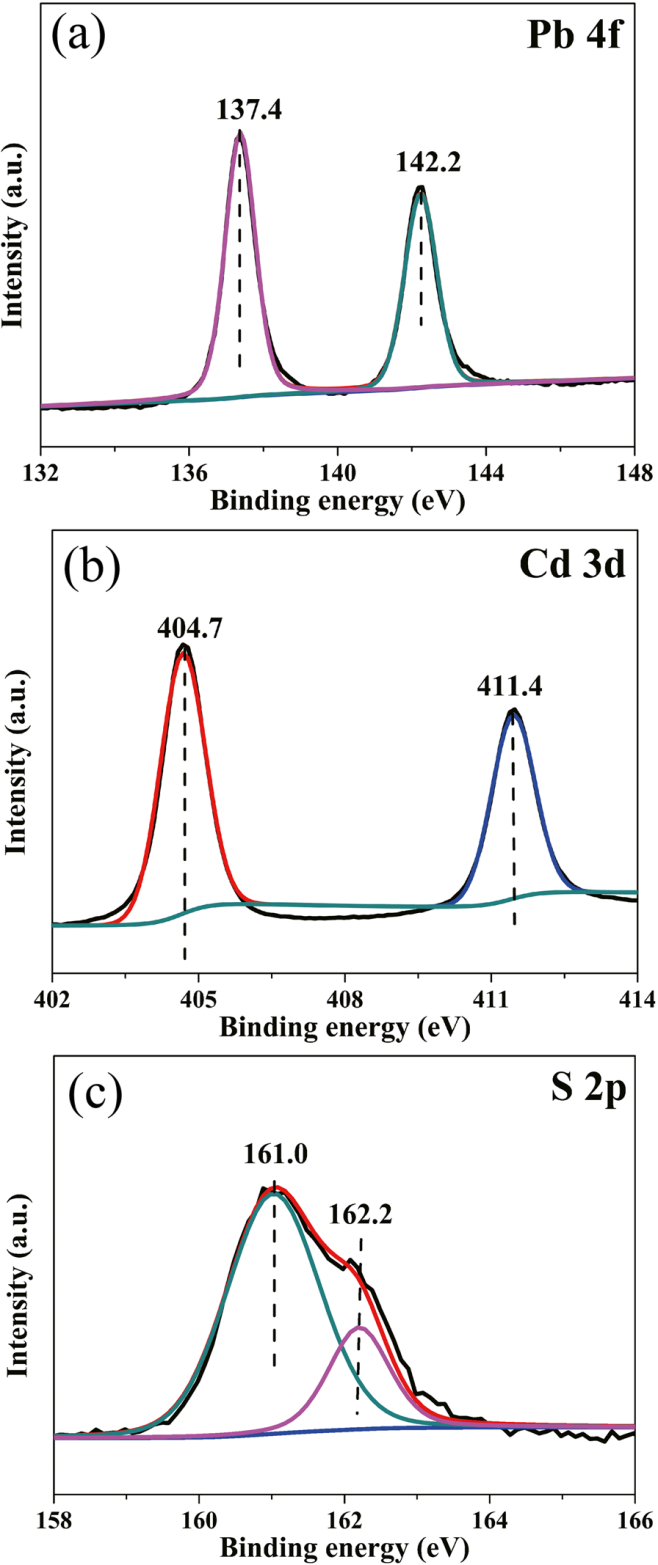
XPS curves of Pb 4f (**a**), Cd 3d (**b**), and S2p (**c**)

The TiO_2_/PbS/CdS sample was further investigated by XPS spectrum shown in Fig. 3. In Fig. 3a, the photoelectron peaks of Pb 4f can be observed at 137.4 and 142.2 eV, which comes from Pb^2+^ ions of PbS QDs [28]. Figure 3b shows the Cd 3d peaks at 404.7 and 411.4 eV, which originates from Cd^2+^ ions of the CdS QDs [33, 34]. In Fig. 3c, the S 2p peaks at 161.0 and 162.2 eV can be assigned to the sulfide of PbS and CdS QDs. Therefore, PbS QDs and CdS QDs are decorated successfully on TiO_2_ nanorod arrays, which are verified by the XRD patterns, EDS, HRTEM, and XPS.

**Fig. 4:**
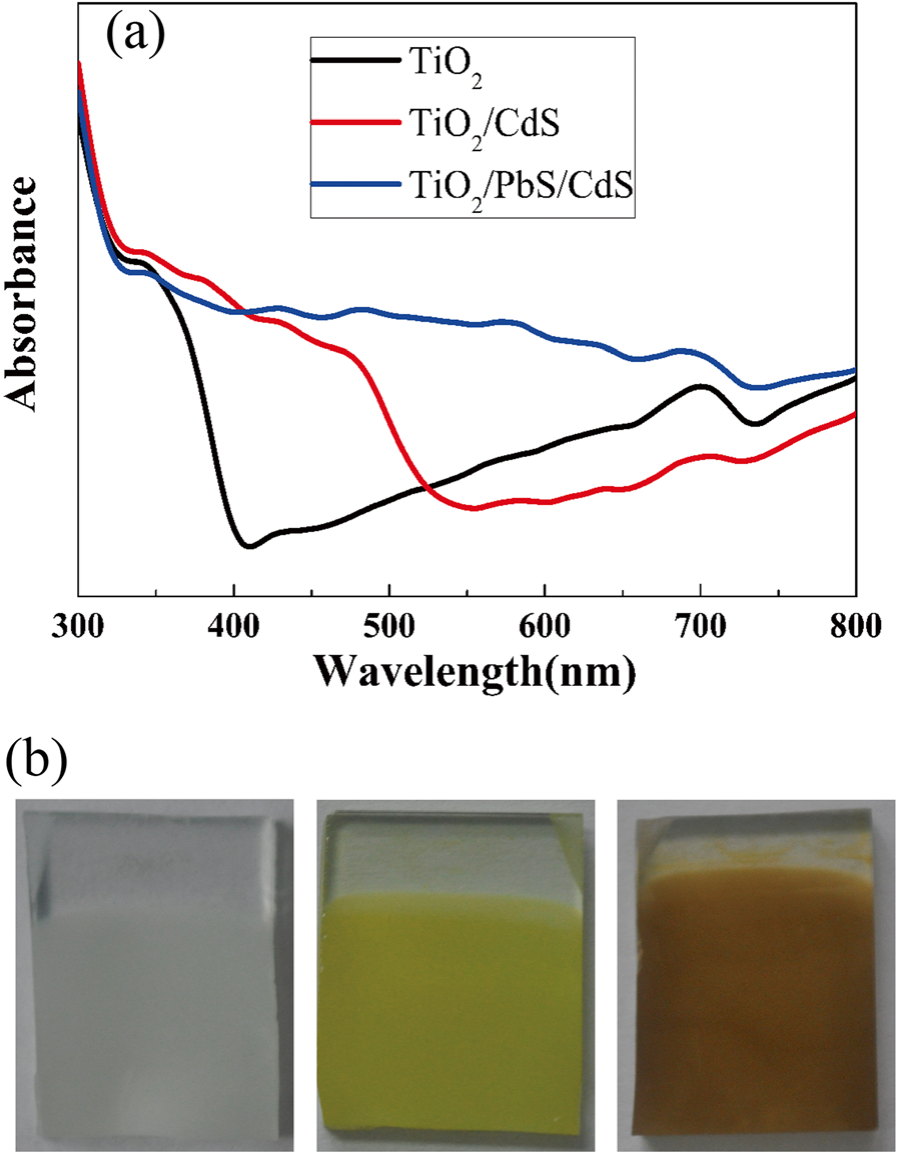
UV-visible absorption spectrum (**a**) and photograph (**b**) of pristine TiO_2_, TiO_2_/CdS, and TiO_2_/PbS/CdS

The optical absorption behavior was illustrated in Fig. 4a. It is obvious that the absorption of TiO_2_/PbS/CdS is enhanced in visible spectrum in comparison with pristine TiO_2_ and TiO_2_/CdS. The photograph of pristine TiO_2_, TiO_2_/CdS, and TiO_2_/PbS/CdS is shown in Fig. 4b. The white color of pristine TiO_2_ is shown to be absorption free in visible light. As CdS is sensitized, the yellow color implies the enhanced absorption in visible light. The brown-black color after sensitized PbS shows the stronger absorption in visible light.

**Fig. 5:**
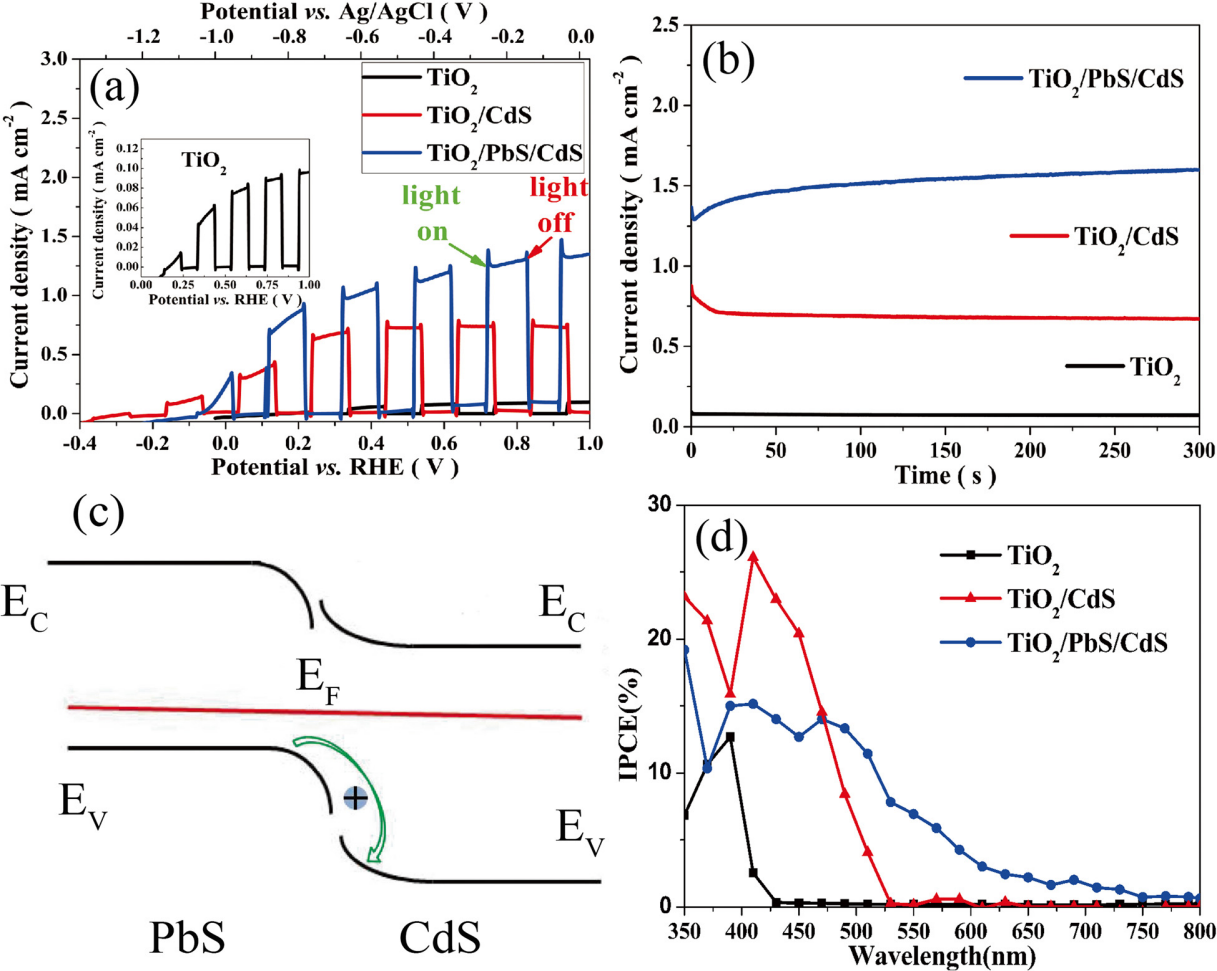
The LSV curves (**a**) of pristine TiO_2_, TiO_2_/CdS, and TiO_2_/PbS/CdS with chopped AM1.5 light illumination. The *I–t* curves (**b**) of pristine TiO_2_, TiO_2_/CdS, and TiO_2_/PbS/CdS at 0.9716 V vs. RHE under illumination. The energy level alignment (**c**) at the interface between PbS and CdS. The IPCE curves (**d**) of pristine TiO_2_, TiO_2_/CdS, and TiO_2_/PbS/CdS measured at 0.9716 V vs. RHE

To characterize the PEC properties of fabricated photoanodes, a three-electrode setup is used, which is shined by 150 W Xeon light source (with AM1.5 filter and 60 mW/cm^2^ power). The details of LSV curves with chopped illumination are shown in Fig. 5a. The current density is only 0.09 mA cm^−2^ for pristine TiO_2_ at 0.9716 V vs. RHE (0 V vs. Ag/AgCl). For TiO_2_/CdS, the current density increases to 0.72 mA cm^−2^ at 0.9716 V vs. RHE. The current density boosts to 1.35 mA cm^−2^ at 0.9716 V vs. RHE for TiO_2_/PbS/CdS, which is ascribed to the enhanced UV-visible absorption. The series of spikes located at the on or off edges of the curves indicate carrier accumulation at the electrode–electrolyte interface and slow oxygen evolving reaction kinetics [16, 20, 35]. Figure 5b shows the *I–t* curves at 0.9716 V vs. RHE under AM1.5 illumination. The current density of TiO_2_/PbS/CdS is highest compared with pristine TiO_2_ and TiO_2_/CdS. However, the TiO_2_/PbS/CdS sample shows more stability than sensitized PbS only [31]. This phenomenon can be attributed to the valence band (VB) offsets between PbS and CdS shown in Fig. 5c, which cause small energy barrier, allowing facile hole transmission from PbS to CdS and preventing the hole accumulation from oxidizing PbS and CdS [36]. From Fig. 5d, the IPCE curve of TiO_2_/PbS/CdS clearly shows the widened UV-visible light absorption region compared to TiO_2_/CdS, even though the IPCE of TiO_2_/CdS is higher than TiO_2_/PbS/CdS under the wavelength of 472 nm. Therefore, the photoanode with TiO_2_/PbS/CdS showed an enhanced PEC performance.

**Fig. 6:**
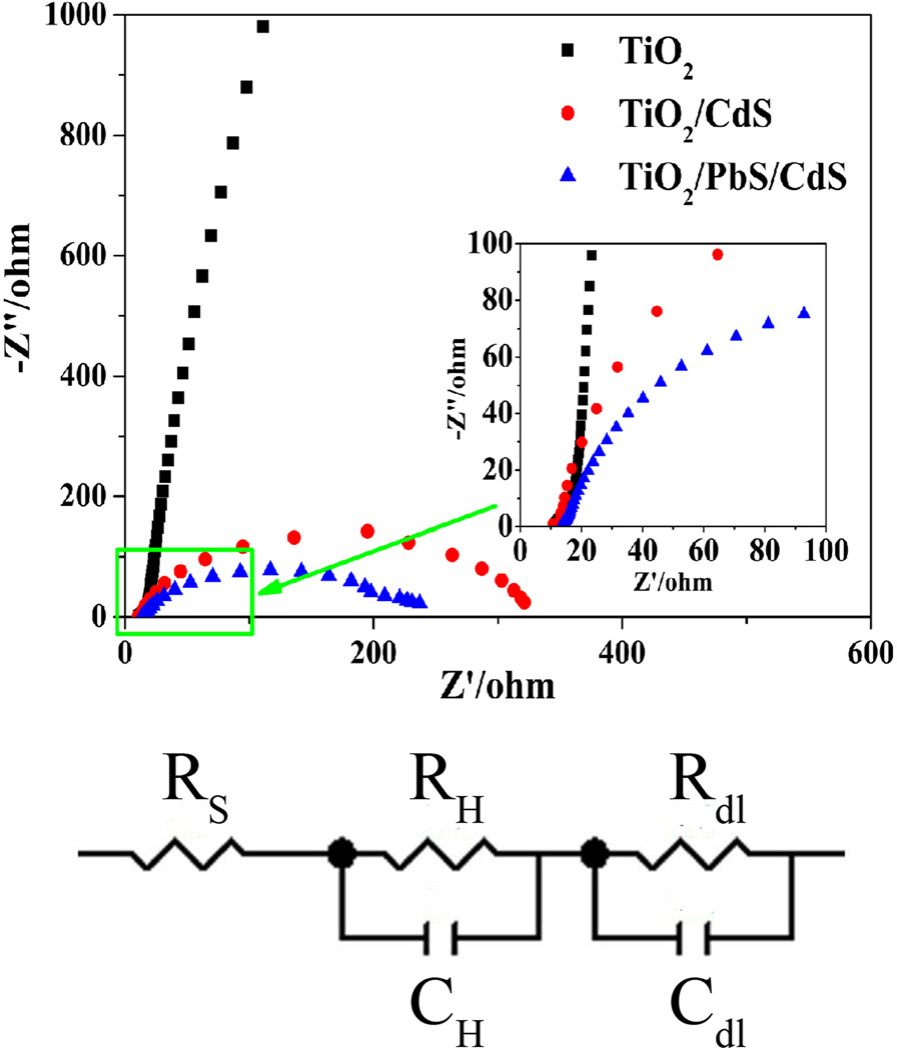
EIS of and equivalent circuit pristine TiO_2_, TiO_2_/CdS, and TiO_2_/PbS/CdS

In order to further investigate the underlying reason for the enhanced PEC performance of fabricated composite photoanodes, the impedance spectroscopy (EIS) of different samples and the equivalent circuit have been shown in Fig. 6. For the equivalent circuit, *R*_*s*_ is the series resistance, *R*_*dl*_ and *C*_*dl*_ are the resistance and capacitance in the semiconductor, and *R*_*H*_ and *C*_*H*_ are the resistance and capacitance at the Helmholtz double layer [37]. As can be seen, the corresponding radii of semicircles (EIS) for pristine TiO_2_, TiO_2_/CdS, and TiO_2_/PbS/CdS decrease sequentially. The smallest radii of the semicircles for TiO_2_/PbS/CdS compared with pristine TiO_2_ and TiO_2_/CdS mean the smallest charge-transfer impedance [38]. Also, the PEC performance can be explained by the value of total resistance (*R*_*t*_ = *R*_*S*_ + *R*_*H*_ + *R*_*dl*_) [39]. The *R*_*t*_ value for pristine TiO_2_ is 5651 Ω cm^−2^, for TiO_2_/CdS is 233 Ω cm^−2^, and for TiO_2_/PbS/CdS is 138 Ω cm^−2^. The lowest *R*_*t*_ results in the highest photocurrent for TiO_2_/PbS/CdS compared with TiO_2_/CdS and pristine TiO_2_.

## Conclusions

The composite photoanode for solar water splitting with sandwiched structures (TiO_2_/PbS/CdS) was prepared by means of facile SILAR method. By introducing PbS QDs between TiO_2_ nanorod arrays and CdS QDs, both the absorption efficiency and stability are improved for the fabricated PEC cell. The highest photocurrent density (1.35 mA cm^−2^ at 0.9716 V vs. RHE) is achieved with TiO_2_/PbS/CdS structure compared to that of pristine TiO_2_ and TiO_2_/CdS. At the same time, the PEC cell of TiO_2_/PbS/CdS is stablest under light illumination. The enhanced performance is attributed to the VB offsets between PbS and CdS, which allow facile hole transmission from PbS to CdS leading to high photon current and photo-corrosion resistance. This structure presents a promising roadmap for high performance and stability devices in solar usage field.
